# Characteristics of a Novel *ATP2B3* K416_F418delinsN Mutation in a Classical Aldosterone-Producing Adenoma

**DOI:** 10.3390/cancers13184729

**Published:** 2021-09-21

**Authors:** Hung-Wei Liao, Kang-Yung Peng, Vin-Cent Wu, Yen-Hung Lin, Shuei-Liong Lin, Wei-Chou Lin, Jeff S. Chueh, on behalf of (TAIPAI) Study Group

**Affiliations:** 1 Chinru Clinic, Taipei 116, Taiwan; lhw898@gmail.com; 2 Department of Internal Medicine, National Taiwan University Hospital, Taipei 110, Taiwan; kangyung@ntu.edu.tw (K.-Y.P.); q91421028@ntu.edu.tw (V.-C.W.); yenhunglin@ntuh.gov.tw (Y.-H.L.); linsl@ntu.edu.tw (S.-L.L.); 3 Department of Pathology, National Taiwan University Hospital, National Taiwan University College of Medicine, Taipei 100, Taiwan; weichou8@ntuh.gov.tw; 4 Department of Urology, College of Medicine, National Taiwan University, National Taiwan University Hospital, Taipei 110, Taiwan

**Keywords:** aldosterone producing adenoma, ATP2B3, K416-F418delinsN mutation, primary aldosteronism

## Abstract

**Simple Summary:**

The *ATP2B3* channel mutation is a rare cause of primary aldosteronism (PA). *ATP2B3* gene mutation leads to the dysfunction of calcium channel that pumps calcium ion out of the cell and accumulates intracellular calcium signal to stimulate aldosterone synthesis. In the present study, we found a novel somatic *ATP2B3* K416_F418delinsN mutation in a PA patient, and proved its functionality by demonstrating aldosterone hyper-function in the mutant-transfected adrenal cell-line. The *ATP2B3* K416_F418delinsN mutation resulted from the deletion from nucleotides 1248 to 1253. The translated amino acid sequence from 416 to 418 as lysine-phenylalanine-phenylalanine was deleted and an asparagine was inserted due to the merging of residual nucleotide sequences.

**Abstract:**

In patients with primary aldosteronism (PA), the prevalence of *ATP2B3* mutation is rare. The aim of this study is to report a novel *ATP2B3* mutation in a PA patient. Based on our tissue bank of aldosterone-producing adenomas (APA), we identified a novel somatic *ATP2B3* K416_F418delinsN mutation. The affected individual was a 53 year-old man with a 4 year history of hypertension. Computed tomography (CT) showed bilateral adrenal masses of 1.6 (left) and 0.5 cm (right) in size. An adrenal venous sampling (AVS) showed a lateralization index (LI) of 2.2 and a contralateral suppression index (CLS) of 0.12; indicating left functional predominance. After a left unilateral adrenalectomy, he achieved partial biochemical and hypertension–remission. This classical adenoma harbored a novel *ATP2B3* K416_F418delinsN somatic mutation, which is a deletion from nucleotides 1248 to 1253. The translated amino acid sequence from 416 to 418, reading as lysine-phenylalanine-phenylalanine, was deleted; however, an asparagine was inserted due to merging of residual nucleotide sequences. The CYP11B2 immunohistochemistry staining demonstrated strong immunoreactivity in this classical adenoma. The *ATP2B3* K416_F418delinsN mutation is a functional mutation in APA, since HAC15 cells, a human adrenal cell line, transfected with the mutant gene showed increased CYP11B2 expression and aldosterone production.

## 1. Introduction

Primary aldosteronism (PA) is originally classified into unilateral hyperaldosteronism and bilateral hyperaldosteronism (BHA) [1]. BHA is mainly related to bilateral idiopathic hyperplasia, which could not be detected by computed tomography (CT) [1,2,3]. Bilateral aldosterone producing adenoma (APA) [4] could be detected as bilateral detectable mass by CT but is a rare finding [1]. The pathogenesis of patients affected by bilateral PA, thought related to BHA, could not be confirmed, because few patients with bilateral PA underwent adrenalectomy, and no such adrenal tissues could be obtained for further investigation. Many somatic mutant genes have been identified that enhance aldosterone secretion; in particular, mutations in *KCNJ5* [5], *CACNA1D* [6], *CACNA1H* [7], *CLCN2* [8], *ATP1A1* [9], and *ATP2B3* [9] genes have been identified. These mutant genes are often related to changes in the function or permeability of ion channels or ion pumps across cell membranes [10].

The aldosterone synthase (CYP11B2) transcription and aldosterone production rely on increased intracellular calcium signaling [10]. After stimulation, zona glomerulosa cells are depolarized and voltage-gated calcium (Ca^2+^) channels on cellular membrane are activated. Subsequently, an influx of extracellular calcium occurs and increases intracellular calcium concentrations and downstream signaling. The ion channel ATP2B3, a Ca^2+^ ATPase type 3, is a protein pump over cellular membrane that exports intracellular calcium out of cells [11]. The mutated *ATP2B3* in aldosterone-producing cells may reduce efflux of calcium ions from cytoplasm, accumulate intracellular calcium, and stimulate aldosterone production [12].

The prevalence of *ATP2B3* mutation in PA is quite low [9]; ranging 1.6% [9]~0.9% [13] from European cohorts. In our previous report, the prevalence of mutated *ATP2B3* gene among our PA patients in Taiwan was 0.5% [14]. In this research, we described a novel mutation of *ATP2B3* K416_F418delinsN in an APA patient who underwent ipsilateral adrenalectomy, and illustrated his clinical characteristics.

## 2. Materials and Methods

### 2.1. Ethics Statement

Ethics approval (approval number 200611031R) was approved by the Institutional Review Committee of National Taiwan University Hospital. Before participating in the study, we obtained a written informed consent form from all participants to collect and study clinical data.

### 2.2. Diagnosis of PA

Based on the Taiwan standard TAIPAI protocol and the consensus on hyperaldosteronism, the referral of patients with hypertension was screened, confirmed and subtyped for PA patients [15,16]. Prior to the PA screening and confirmation test, all original antihypertensive drugs were discontinued for at least 21 days. We prescribed doxazosin and/or diltiazem during the evaluation phase as needed to control markedly hypertension. The diagnosis of PA in patients with hypertension was according to the abnormal hypersecretion of aldosterone and met the criteria [16,17,18,19,20,21,22,23,24,25,26].

### 2.3. Genomic DNA Extraction

Tumoral and adjacent adrenal tissue’s genomic DNA was extracted by using QIAamp DNA mini kit (Qiagen, Hilden, Germany); genomic DNA from peripheral whole blood was extracted by using Blood DNA Isolation Kit (Geneaid Biotech; New Taipei City, Taiwan) according to the manufacturer’s instructions.

### 2.4. ATP2B3 Gene Sequencing

The coding regions containing well- characterized mutations of *ATP2B3* gene were amplified and sequenced by using gene-specific primers and the BigDye^®^ Terminator v3.1 Cycle Sequencing Kit (Applied Biosystems Inc., Foster City, CA, USA) on the 3730 DNA Analyzer (Applied Biosystems, Foster City, CA, USA). The primers of polymerase chain reaction (PCR) used to amplify fragments for *ATP2B3* direct sequencing followed that from a previous report [21] (forward CCTGGGCTGTTTATCCTGAA, reverse CCCCAGTTTCCGAGTCTGTA). The sequences analysis was performed by using the DNAStar Lasergene SeqMan Pro 7.1.0 software (DNAStar Inc., Madison, WI, USA).

### 2.5. Immunohistochemistry of Resected Tissues

The CYP11B2 and 17α-hydroxylase (CYP17A1) mouse monoclonal antibody, CYP11B1 rat monoclonal antibody (generous gifts from Professor Celso Gomez-Sanchez [27]), and HSD3B mouse monoclonal antibody (Abnova, Taipei, Taiwan) were used for immunohistochemistry (IHC) [28]. The polymerized horseradish peroxidase (HRP)-anti-mouse conjugate method (Novolink; Novocastra Laboratories Ltd., Newcastle Upon Tyne, UK) was used to stain sections of paraffin-embedded adrenal tumors and surrounding tissues according to the manufacturer’s instructions [14]. The images were captured by Olympus BX51 fluorescence microscope combined with Olympus DP72 camera and cell Sens Standard 1.14 software (Olympus, Hamburg, Germany) was used for image analysis.

### 2.6. Culture of Cell Line

We used HAC15 cell, a human adrenocortical cell line, which express aldosterone synthase, CYP11B2, and secrete aldosterone for aldosterone production study. The HAC 15 cell line was obtained from generous Dr. Silvia Monticone [29]. HAC15 cells were cultured in HAC15 complete media containing DMEM:F12 (1:1) supplemented with 10% cosmic calf serum, 1× ITS, 1% penicillin–streptomycin and 100 μg/mL primocin at 37 °C. We used humidified incubator with 5% CO_2_ to incubate the cultured cells, as previously reported [17].

### 2.7. Plasmid and Transfection

We used PCR-assisted, site-directed mutagenesis for the plasmids, expressing the wild-type and mutant *ATP2B3* genes and cloning into the pIRES-GFP-puro vector. PCR-based direct sequencing confirmed that the mutation was successfully cloned into the vector. Moreover, 3 × 10^6^ cells HAC15 cells were transiently transfected with 3 μg pIRES-GFP empty vector, pIRES-GFP-wild-type *ATP2B3* or pIRES-GFP *ATP2B3* K416_F418delinsN using the Amaxa Cell Line Nucleofector Kit R (Lonza, Cologne, Germany) and the Nucleofector I (program X-005), according to the instructions of the manufacturer. After transfection, we seeded the HAC15 cells with a density of 1 × 10^6^ cells/well into a 6-well plate. Furthermore, 72 h after the transfection, the cells and culture supernatants were harvested for Western blot analysis and aldosterone measurement.

### 2.8. Western Blot Analysis

Using RIPA buffer (50 mM Tris base pH 8, 150 mM NaCl, 1% NP40, 0.10% SDS) containing a protease inhibitor (Roche Diagnostics, Indianapolis, IN, USA), proteins were isolated from whole cell extracts. After we centrifuged the cell lysates, the supernatants were mixed with 3× sample buffer (30% glycerol, 15% 2-mercaptoethanol and 1% bromophenol blue). The proteins were separated through 12% SDS-PAGE gels and electrophoretic transferred to PVDF membranes. The membranes were then blocked by incubating in the BlockPRO™ blocking buffer (Visual Protein Biotechnology, Taipei, Taiwan) for 1 h blocking buffer containing anti-CYP11B2 mouse monoclonal antibody (a kind gift from Professor Celso Gomez-Sanchez) and anti-GAPDH antibody were incubated overnight at 4 °C. Extensive washing was conducted by Tris-buffered saline containing 0.1% Tween-20 (TBST) buffer. We further incubated the transfer membranes in blocking buffer that contained HRP-conjugated secondary antibodies for 1.5 h. Subsequently, the membranes were washed with TBST three times. Enhanced chemiluminescent reagent (Thermo Scientific, Rockford, IL, USA) was applied at a ratio of 1:1. Reagents for Chemiluminescence detection (Millipore, Billerica, MA, USA) were used to detect protein levels, and UVP Biospectrum 810 imaging system (Ultra Violet Products Ltd., Cambridge, UK) was used for visualization. We quantified protein expression in each sample by using UVP software (Ultra Violet Products Ltd., Cambridge, UK). A densitometry analysis of each protein band was normalized to GAPDH levels and expressed as a relative fold change when compared with the non-transfected control.

### 2.9. Analysis of Aldosterone

The culture supernatants were collected 72 h after cells transfected with *ATP2B3* K416_F418delinsN mutant or wild type plasmids to measure aldosterone concentration (ALDO-RIACT RIA kit, Cisbio Bioassays, Codolet, France) [28].

### 2.10. Statistical Analysis

The experimental differences between the transfected gene groups were analyzed by one-way ANOVA with post hoc least significant difference test (LSD) tests. A two-sided *p* value < 0.05 was defined as statistically significant. All of the statistical analyses were performed using IBM SPSS statistics version 19 (IBM Corp, Armonk, NY, USA) software.

## 3. Results

### 3.1. Identifying the ATP2B3 K416_F418delinsN Gene and Demographics of the Specific Patient

In DNA samples extracted from APA tumor tissues, we identified the mutant *ATP2B3* gene in a left adrenal adenoma. The affected individual was a 53 year-old man. He had a history of hypertension for more than 4 years and presented with uncontrollable hypertension and hypokalemia (2.8 mEq/L) for further survey. After the standardized screening and confirmation tests, his PA was diagnosed. A computer tomography scan showed a left 1.6 cm and a right 0.5 cm adrenal masses. An adrenal venous sampling (AVS) showed functionally predominant aldosterone hypersecretion over his left adrenal gland (Table 1). The lateralization index (LI) was 2.2 and the contralateral suppression index (CLS) was 0.12. The result of the iodine-131 6-beta-iodomethyl-19-norcholesterol adrenal scintigraphy (NP-59 scan) was also compatible with a functional left adrenal adenoma. After a left adrenalectomy, his hypertension achieved clinical partial remission, with reduced doses of anti-hypertensive medications (Table 1). In the postoperative biochemical tests, hypokalemia was resolved; however, the aldosterone to renin ratio (ARR) remained high. Therefore, biochemical outcome reached only partial success [30].

**Figure 1:**
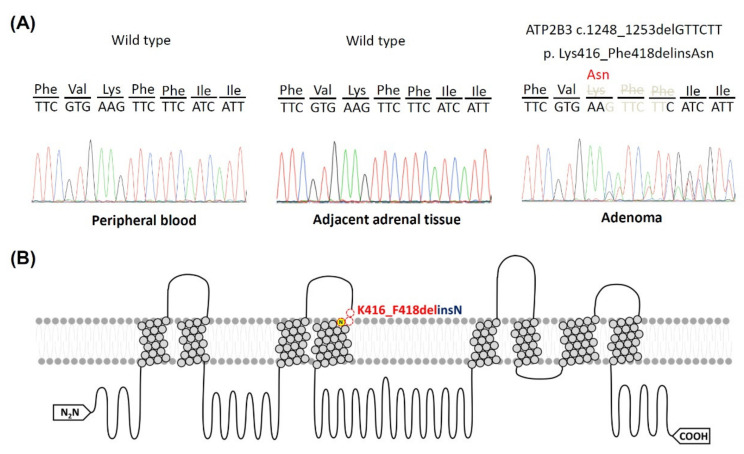
A novel *ATP2B3* K416-F418delinsN mutation in classical APA. (**A**) The deletion at nucleotide 1248 to 1253 as GTTCTT in the *ATP2B3* K416-F418delinsN gene was identified in a patient with APA in the resected adrenal adenoma. The amino acid residue 416 to 418 of ATP2B3 protein was substituted from lysine (Lys) and 2 phenylalanine (Phe) to asparagine (Asn) insertion. The letters for nucleotide bases represented as following: C, cytosine; G, guanine; T, thymine; (**B**) the protein secondary structure of mutant ATP2B3 channel was demonstrated. The yellow circle N represented insertion of amino acid, asparagine. The model was based on Protter software application [31] (http://wlab.ethz.ch/protter/start/ (accessed on 22 July 2021) [32]).

This novel *ATP2B3* K416_F418delinsN somatic mutation was identified only in the resected adrenal tissue, but not in peripheral blood cells and adjacent adrenal tissue. The *ATP2B3* K416_F418delinsN somatic mutation resulted from the deletion at nucleotide 1248 to 1253 as GTTCTT (Figure 1). The resulting amino acid sequence showed the deletion of 416 lysine (Lys, K), 417 phenylalanine (Phe, F) and 418 phenylalanine (Phe, F) due to the deletion of the nucleotide from 1248 to 1253 (Figure 1). However, a new amino acid, asparagine, was found due to merged nucleotides from 1246, 1247 and 1254. Therefore, the original amino acid sequence as lysine-phenylalanine- phenylalanine was not encoded, but instead a new amino acid, asparagine, was inserted.

### 3.2. The Immunochemistry Staining of CYP11B2 on Excised Adrenal Tissue

**Figure 2:**
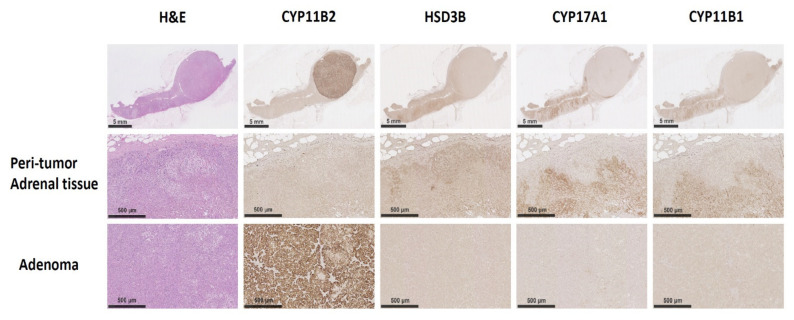
The immunohistochemistry staining in unilateral PA patients with the *ATP2B3* K416-F418delinsN mutations. The CYP11B2 and other steroidogenesis related enzyme IHC staining was conducted. The CYP11B2 immunoactivity was stained intensely within the adenoma, but did not stain in the adjacent adrenal gland tissue. Of note, HSD3B, CYP17A1 and CYP11B1 did not observed with adenoma but scattered in the residual adrenal gland tissue. Scale bar represented 500 μm.

The gross slide section showed a well-demarcated and easily identified classical APA in the excised adrenal gland. The steroid 18-hydroxylase (CYP11B2; aldosterone synthase) IHC staining showed intense density within that compact zona glomerulosa (ZG)-like adenoma (Figure 2). No CYP11B2 IHC staining was found in the adjacent adrenal cortical tissues. For other steroidogenesis related enzymes, such as 3β-Hydroxysteroid dehydrogenase (HSD3B), 17α-hydroxylase (CYP17A1), and 11β-hydroxylase (CYP11B1), their IHC staining was not enhanced in the adenoma, but was observed in the adjacent adrenal gland tissues.

### 3.3. The Aldosterone Synthase of ATP2B3 K416-F418delinsN Mutation

**Figure 3:**
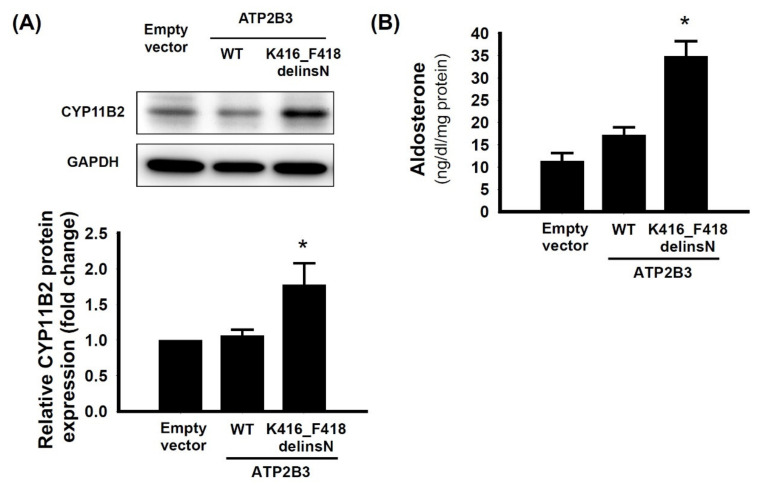
The CYP11B2 synthase and aldosterone production in the HAC 15 cells with transfected *ATP2B3* K416_F418delinsN. The aldosterone synthase, CYP1B2, expression and supernatant aldosterone levels were analyzed at 72 h after plasmid transfection. (**A**) The cells transfected with *ATP2B3* K416_F418delinsN had increased CYP11B2 protein expression in compared with the cells transfected with wild type cells; (**B**) The aldosterone levels of supernatant in the culture cells also increased in transfected group compared with the wild type cells. The data are presented as the means ± SD of three independent experiments in the transfected wild type and mutant cells. * *p* < 0.05 represented significant difference. The uncropped Western Blot image can be found in Figure S2.

We transfected the mutant *ATP2B3* K416_F418delinsN gene to HAC15 cells and investigated the physiological effects of this novel mutation. The expression of CYP11B2 in mutant-gene transfected cells was increased compared to that of control cells transfected with empty vector or wild type *ATP2B3* (Figure 3). The aldosterone levels in the supernatant of the culture medium were higher in mutant-gene transfected cells compared to that from control vector or the wild type cells. Thus, the deletion of amino acid expression of lysine, and two phenylalanine residues from 416 to 418, along with an insertion of asparagine, showed a gain-of-function mutation at *ATP2B3* channel for aldosterone over-production.

## 4. Discussion

In this study, we found a novel functional somatic *ATP2B3* K416_F418delinsN mutant gene from our APA tissue bank. The histopathological examination showed a well-defined compact, ZG-like classical adenoma and intense immunoreactivity to CYP11B2 staining. The cells transfected with the indicated mutant gene demonstrated increased CYP11B2 expression and elevated aldosterone levels in the culture supernatant when compared with that of the wild-type cells. Moreover, we have used Sanger sequencing to confirm that there were no other conventional and well-characterized aldosterone-driving gene mutations, including *KCNJ5, ATP1A1, CACNA1D,* and *CTNNB1,* in the adenoma harboring with *ATP2B3* K416_F418delinsN mutation (Figure S1).

### 4.1. Calcium Channel and Somatic Mutations in APA

The transcription of *CYP11B2* could be activated by intracellular calcium signaling [33]. Consistently increased cytoplasmic calcium concentration or stimulation may lead to the excessive production of aldosterone [34], which is the main mechanism of PA. Mutant KCNJ5, and CLCN2 ion channels are involved in cellular membrane depolarization and subsequently activate voltage-gated Ca^2+^ channels to increase Ca^2+^ influx [34,35] into cytoplasm. However, ion channels of CACNA1D and CACNA1H control the entry of extracellular calcium, and enhance the Ca^2+^ permeability with their mutant voltage-gated Ca^2+^ channels. Unlike other channels that increase intracellular calcium by affecting Ca^2+^ entry, the mutated ATP2B3 ion channel reduces Ca^2+^ export from cytoplasm. Thus, there are two possible ways to increase stimulating signaling in ATP2B3 ion channel [11]: (1) reduce clearance of intracellular calcium and directly stimulating *CYP11B2* transcription, and (2) accumulation of cation leads to Na^+^ influx and subsequently depolarizes the cellular membrane and activates a downstream reaction [11].

### 4.2. Mutant ATP2B3 and APA

ATP2B3 ion channel belongs to plasma membrane Ca^2+^ ATPase (PMCA) transporter. The *ATP2B3* mutant APAs have higher serum aldosterone levels and lower potassium levels compared to other mutant genes related to APA [36].

Our index patient with *ATP2B3* K416_F418delinsN had uncontrollable hypertension and hypokalemia at presentation. In accordance with our finding, most identified *ATP2B3* mutation in APA were expressed mainly in ZG-like cells [37]. The IHC showed condensed CYP11B2 staining in the adenoma, but not in the peri-tumoral region. Therefore, the source of excess aldosterone could arise from the classical adenoma, in concordance with the location of the mutant gene. We have also confirmed that the adrenal tissue adjacent to the adenoma, besides that from white blood cells, carried wild-type *ATP2B3* gene by using Sanger sequencing (showed in the Figure 1A and Table S1).

### 4.3. Bilateral Asymmetric Manifestations of the APA

This patient had bilateral adrenal masses, and CT showed a larger mass on the left side. The LI of AVS was 2.2 (>2.0) [38], indicating a left functional predominance. The CLS was 0.12 (<1.0) [39,40], indicating the suppression of the right adrenal gland. The preoperative diagnosis was unilateral PA over the left adrenal gland. However, 1 year after left unilateral adrenalectomy, blood pressure control only achieved partial success. The serum potassium level was normalized from 2.8 to 4.0 mEq/L. However, the ARR after unilateral adrenalectomy remained high. According to the PASO consensus, the biochemical outcome achieved only partial success [30]. Thus, from the functional and clinical responses, this patient with *ATP2B3* K416_F418delinsN mutation may have abnormal contralateral adrenal gland and bilateral asymmetric aldosterone secretion. However, before the left total adrenalectomy, the aldosterone secretion from the right adrenal gland was suppressed by the left functional adenoma initially; once the patient underwent left adrenalectomy, the suppression from the left adrenal was gone, and the aldosterone secretion from the right adrenal gland, probably in the form of multiple aldosterone-producing micronodules (mAPM) or APA, may take over and contribute to the bilateral asymmetric disease, and led to his incomplete blood pressure and biochemical recovery.

### 4.4. Clinical Implication and Study Limitations

Different mutant genes of PA have specific pathophysiological, clinical and biochemical manifestations [36]. Identifying functional genes in PA would help physicians determine the course of the disease and make decision on treatment and follow-up. Patients with *ATP2B3* mutant APA have obvious aldosterone and potassium abnormalities [36]. Most reported that APA harboring *ATP2B3* mutant was unilateral APA. Our finding of this new mutation will help researchers in this field to incorporate this mutation in their future routine screening of the possible mutation spots, and the actual prevalence of this novel mutation will be further assured. Although left APA was confirmed by the AVS and resected, the pathophysiological characteristics in the contralateral adenoma could not be obtained. Furthermore, we have only one APA patient harboring this novel *ATP2B3* K416_F418delinsN gene and could not conclude a general relationship between the genotype and phenotype.

## 5. Conclusions

In conclusion, we identified a patient with an APA harboring a novel *ATP2B3* K416_F418delinsN somatic mutation. He became a partial hypertension-remission and biochemical success after unilateral adrenalectomy. HAC15 cells harboring this *ATP2B3* K416_F418delinsN somatic mutation increased CYP11B2 synthesis and aldosterone production. The immunohistochemistry staining showed a compact and well demarcated ZG-like adenoma, with intense CYP11B2 expression. Thus, this novel somatic mutation of *ATP2B3* K416_F418delinsN functionally increased aldosterone secretion, and it also showed a distinct histopathologic pattern, as well as an important clinical signature.

## Figures and Tables

**Table 1:** The basal characteristics of the uPA patient with adenoma harboring *ATP2B3* K416-F418delinsN deletion patient.

Variables	*ATP2B3* K416_F418delinsN Mutation
Age (years old)	53
Sex	male
Body weight (kg)	75
BMI (kg/m^2^)	25.95
CT mass size (cm)	Left: 1.6; Right: 0.5
AVS (aldosterone, ng/dL)/cortisol (μg/dL)	
CLS	0.12
LI	2.2
NP-59	Bilateral adrenal gland hyperfunction with left side predominance
Hypertension duration (years)	4
SBP (mm Hg)	197
SBP 12 mon	158
DBP (mm Hg)	92
DBP 12 mon	88
Aldosterone level (ng/dL) ^†^	59.3
PRA (ng/mL/hr) ^†^	0.55
ARR(ng/dL per ng/mL/h)	107.82
K (mEq/L) ^†^	2.8
At 12 months after adrenalectomy	
Aldosterone level	30.5
PRA	0.09
K (mEq/L) ^†^	4.0
ARR (ng/dL per ng/mL/h)	338.33
Clinical success	partial success ^§^
Biochemical success	partial success

Abbreviations: ARR, aldosterone renin ratio; BMI, body mass index; CLS: contralateral suppression index; DBP, diastolic blood pressure; K, potassium; LI, lateralization index; NP-59, The iodine-131 6-beta-iodomethyl-19-norcholesterol adrenal scintigraphy; PA, primary aldosteronism; PRA, plasma renin activity; SBP, systolic blood pressure. ^§^ Partial success represents that the same blood pressure as before surgery but with less antihypertensive medication or decreased BP by the same or less antihypertensive medication. ^†^ Obtained after withholding drugs that interfere with the renin-angiotensin system.

## Data Availability

The data are all included in the manuscript or can be acquired from the corresponding author.

## Supplementary Materials

The following are available online at https://www.mdpi.com/article/10.3390/cancers13184729/s1, Figure S1. Sanger sequencing analysis of tumor DNAs for conventional and well-characterized aldosterone-driving gene mutations, including *KCNJ5*, *ATP1A1*, *CACNA1D*, and *CTNNB1*, in the adenoma harboring with *ATP2B3* K416_F418delinsN mutation, Figure S2. Uncropped Western Blot images from Figure 3 in the main text, Table S1. Primers used for Sanger sequencing.
