# Supplementary material for: Characteristics of a Novel ATP2B3 K416_F418delinsN Mutation in a Classical Aldosterone-Producing Adenoma
Source: Cancers (Basel). 2021 Sep 21;13(18):4729. doi: 10.3390/cancers13184729 (PMC8472399; doi:10.3390/cancers13184729)
Supplement: Supplementary file 1 [file cancers-13-04729-s001.zip › cancers-1371352-supplementary.pdf]

Supplementary Materials: Characteristics of a Novel *ATP2B3* K416_F418delinsN Mutation in a Classical Aldosterone-Producing Adenoma

Hung-Wei Liao, Kang-Yung Peng, Vin-Cent Wu, Yen-Hung Lin, Shuei-Liong Lin, Wei-Chou Lin and Jeff S. Chueh, on behalf of (TAIPAI) Study Group

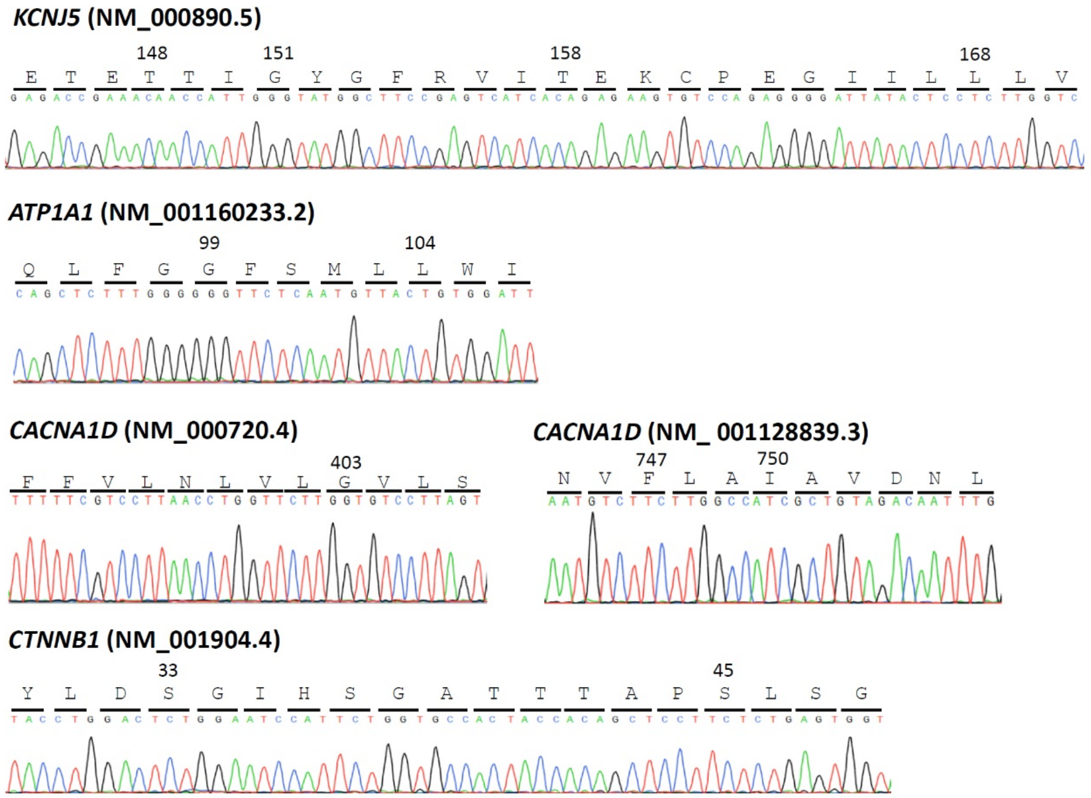

Figure S1. Sanger sequencing analysis of tumor DNAs for conventional and well-characterized aldosterone-driving gene mutations, including *KCNJ5*, *ATP1A1*, *CACNA1D*, and *CTNNB1*, in the adenoma harboring with *ATP2B3* K416_F418delinsN mutation.

CYP11B2

Original data in this manuscript

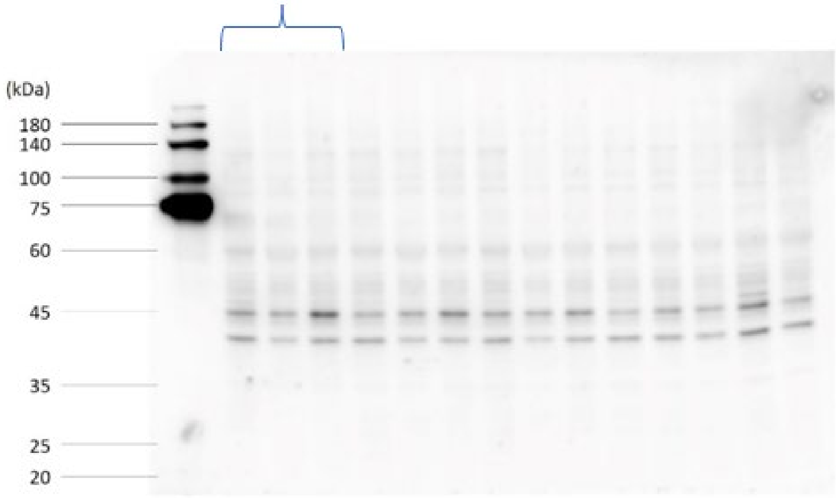

GAPDH

Original data in this manuscript

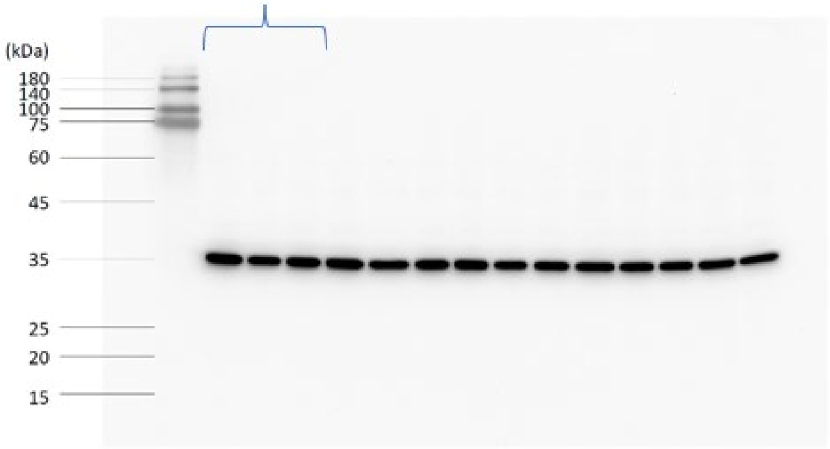

Figure S2. Uncropped Western Blot images from Figure 3 in the main text.

Table S1. Primers used for Sanger sequencing.

ATP2B3 (NM_001001344.2)			Detectable Mutations	Base Pair
ATP2B3-Exon 8 (1)	Forward	CCTGGGCTGTTTATCCTGAA	p.Val424_Leu425del<br>p.Leu425_Val426del<br>p.Val426_Val427del	416
	Reverse	CCCCAGTTTCCGAGTCTGTA		
CACNA1D **(NM_000720.4)**			**Detectable Mutations**	**Base Pair**
CACNA1D-Exon 8 (2)	Forward	GCCTTGATGACTCTGTGTG	p.Gly403Arg	453
	Reverse	CCAGCAAAGCTTGTGTGGT	p.Gly403Asp	
CACNA1D **(NM_001128839.3)**			**Detectable Mutations**	**Base Pair**
CACNA1D-Exon 16 (2)	Forward	TTTACTTCTGTAGACTGTCC TTTTA	p.Phe747Leu	367
	Reverse	ACACGTGACTCCCACTCTCAGC	p.Ile750Met	
CTNNB1 **(NM_001904.4)**			**Detectable Mutations**	**Base Pair**
CTNNB1-Exon 3 (3)	Forward	CATTCTGCTTTTCTTGGCTGTC	p.Ser33Cys<br>p.Gly34Arg<br>p.Ser45Phe<br>p.Ser45Pro<br>p.Ser45Cys	483
	Reverse	GCTATTACTCTCTTTTCTTCCC		
ATP1A1 **(NM_001160233.2)**			**Detectable Mutations**	**Base Pair**
ATP1A1-Exon 4 (1)	Forward	TTCCTTGGGCCTATTGTTTG	p.Gly99 Arg	487
	Reverse	GTGGGAGACAAAGACGGAGA	p.Leu104Arg	

References

1. Wu, V.-C.; Huang, K.-H.; Peng, K.-Y.; Tsai, Y.-C.; Wu, C.-H.; Wang, S.-M.; Yang, S.-Y.; Lin, L.-Y.; Chang, C.-C.; Lin, Y.-H.; et al. Prevalence and clinical correlates of somatic mutation in aldosterone producing adenoma-Taiwanese population. *Sci. Rep.* **2015**, *5*, 11396, doi:10.1038/srep11396.

2 Fernandes-Rosa, F.L.; Williams, T.A.; Riester, A.; Steichen, O.; Beuschlein, F.; Boulkroun, S.; Strom, T.M.; Monticone, S.; Amar, L.; Meatchi, T.; et al. Genetic Spectrum and Clinical Correlates of Somatic Mutations in Aldosterone-Producing Adenoma. *Hypertension* **2014**, *64*, 354–361, doi:10.1161/hypertensionaha.114.03419.

3. Teo, A.; Garg, S.; Shaikh, L.H.; Zhou, J.; Frankl, F.E.K.; Gurnell, M.; Happerfield, L.; Marker, A.; Bienz, M.; Azizan, E.A.; et al. Pregnancy, Primary Aldosteronism, and AdrenalCTNNB1Mutations. *New Engl. J. Med.* **2015**, *373*, 1429–1436, doi:10.1056/nejmoa1504869.
